# Association Between Physical Activity and Proximity to Physical Activity Resources Among Low-Income, Midlife Women

**Published:** 2006-12-15

**Authors:** Stephanie B Jilcott, Kelly R Evenson, Barbara A Laraia, Alice S Ammerman

**Affiliations:** University of North Carolina at Chapel Hill, Department of Nutrition, Schools of Public Health and Medicine, and the UNC-CH Center for Health Promotion and Disease Prevention; University of North Carolina at Chapel Hill, Department of Epidemiology, School of Public Health, Chapel Hill, NC; University of North Carolina at Chapel Hill, Department of Nutrition, Schools of Public Health and Medicine, and the Carolina Population Center, Chapel Hill, NC; University of North Carolina at Chapel Hill, Department of Nutrition, Schools of Public Health and Medicine, and the UNC-CH Center for Health Promotion and Disease Prevention, Chapel Hill, NC

## Abstract

**Introduction:**

The association between levels of physical activity and perceived and objectively measured proximity to physical activity resources is unclear. Clarification is important so that future programs can intervene upon the measure with the greatest association. We examined correlations between perceived and objectively measured proximity to physical activity resources and then examined associations between both measures of proximity and objectively measured physical activity.

**Methods:**

Participants (n = 199) were underinsured women from three counties in southeastern North Carolina. Perceived proximity to physical activity resources (e.g., parks, gyms, schools) was measured using surveys. Objectively measured proximity included geographic information systems road network distance to the closest resource and existence of resources within 1- and 2-mile buffers surrounding participants' homes. To examine the association between proximity to resources and activity, the dependent variable in multiple linear regression models was the natural logarithm of accelerometer-measured moderate to vigorous physical activity in minutes per day.

**Results:**

Pearson correlation coefficients for perceptions of distance and objectively measured distance to physical activity resources ranged from 0.40 (gyms, schools) to 0.54 (parks). Perceived distance to gyms and objective number of schools within 1-mile buffers were negatively associated with activity. No statistically significant relationships were found between activity and perceived or objectively measured proximity to parks.

**Conclusion:**

Results indicate the need for both individual and environmental intervention strategies to increase levels of physical activity among underinsured, midlife women. More work is needed to determine the most effective strategies.

## Introduction

Physical activity (PA) is an important health behavior for chronic disease control and prevention (1). Residents of the rural United States and southern United States are more likely to be inactive than those in other areas of the country (2), and individuals from racial and ethnic minority groups are less likely to have met recommendations for moderate- or vigorous-intensity leisure-time PA compared with the national average (3). Access to PA facilities and resources can support individuals' efforts to be physically active. Thus, it is important to study the relationship between access to PA resources and moderate- to vigorous-intensity PA (MVPA) in rural and semiurban communities with large percentages of low-income households and individuals from racial and ethnic minorities. People in these communities may have less geographic and economic access to supportive PA resources (4-6), potentially exacerbating existing health disparities.

Perceived (7,8) and objectively measured proximity (9-11) to PA resources have been associated with adult PA levels in several studies. It is likely that the two measures of proximity are correlated, yet research findings on the correlation conflict. Sallis et al (9) found no significant correlation between individuals' perceptions of the convenience of PA facilities and objectively measured density of PA facilities. However, another study found a moderate correlation between self-reported distance and geographic information systems (GIS) network distance to a community trail (10). Therefore, the first aim of this study was to examine the agreement between perceived and objectively measured proximity to PA resources among low-income, midlife (aged 40 to 64 years) women. PA resources included public parks, gyms and recreation centers, and public schools, because these are resources that midlife women are likely to use (12,13).

Measuring PA objectively is important to avoid bias due to inaccurate participant recall or social desirability (14). King et al found that residents who reported living within walking distance to a park, trail, or department, discount, or hardware store had higher numbers of steps — objectively measured by a pedometer — than people who reported not living within walking distance of one of these locations (15). Another recent study revealed that GIS-measured proximity to a golf course and to a post office was associated with more pedometer-measured steps (11). Three additional studies of the association between perceived and objectively measured environmental variables and objectively measured PA (16-18) suggest that residents of pedestrian-oriented neighborhoods had significantly more minutes of objectively measured PA. However, we found no studies examining the relationship between objectively measured distance to and density of PA resources and accelerometer-measured PA among midlife women.

Little is known about the influence of perceived proximity to PA resources on MVPA compared with objectively measured proximity. If perceived proximity is more strongly associated with MVPA, then intervening to increase awareness of existing community resources may be most effective. If objectively measured proximity is more influential, then environmental interventions are likely needed. Therefore, the second aim of this study was to examine the association between objectively measured MVPA and perceived and objectively measured proximity to PA resources. Because perceived proximity includes a combination of an individual's awareness that the resource exists and the actual distance to the resource, as well as perceptions about barriers or facilitators to resource use (e.g., cost, safety), we hypothesized that perceived proximity to PA resources would be more strongly associated with MVPA than objectively measured proximity.

## Methods

This study was a cross-sectional analysis using data from a clinic-based, randomized trial conducted by the North Carolina WISEWOMAN program, part of a project funded by the Centers for Disease Control and Prevention (CDC) to help reduce cardiovascular disease risk in under- and uninsured, midlife women; details of this trial are provided elsewhere (19,20). The 12-month intervention included individual and group sessions with a health counselor, contacts with a community health advisor, mailings, and community resource tools designed to increase women's awareness and use of local PA and nutrition resources (20). During the WISEWOMAN study, data on perceived proximity to PA resources and quantitative data on PA levels were collected; these data were used in combination with GIS data to assess the relationship between perceived and objectively measured distances to PA resources and objectively measured PA levels. A subset of all study participants was included in the current analyses; the subset included participants from three southeastern North Carolina counties (New Hanover, Brunswick, and Pender). All perceived proximity and accelerometer data were collected at participants' 12-month follow-up visits. This study was approved by the School of Public Health Institutional Review Board on Research Involving Human Subjects at the University of North Carolina at Chapel Hill.

### Setting

The WISEWOMAN trial, conducted from May 2003 through December 2004, was based in one community health center in Wilmington, NC, a moderately sized town surrounded by smaller coastal and agricultural areas. At the time of the study, the census bureau population estimate for Wilmington was 93,292, and estimates for the least to most populated surrounding incorporated places where participants lived ranged from 483 to 5192 people (21). 

### Perceived proximity to resources

For the first measure of proximity, three items were used to assess perceived distance to the closest PA resource: "How close to your home, in miles, is the closest [school; gym or recreation center; park]?" Three continuous perceived distance variables were created.

For the second measure of proximity, neighborhood was defined as a 10-minute drive from home, and three items measured perceived existence of PA resources in the participant's neighborhood: "Is there a [school; gym or recreation center; park or trail] where you could exercise in your neighborhood?" Response options were yes, no, and don't know, and three dichotomous variables were created to indicate presence (yes) or absence (no, don't know) of a resource in the neighborhood. Although survey measures were not validated, previous work has demonstrated that similar items have moderate to large reliability (22,23).

### GIS database

The following locations were geocoded in ArcGIS Version 9.1 (Environmental Systems Research Institute, Redlands, Calif): participants' home addresses, parks, gyms and recreation centers, and public schools that allowed the public to use PA facilities. Participants' home addresses were mapped as points, and 10% of all participants' addresses with the lowest match scores were checked against the appropriate road network file. (The match score indicates how closely elements of the address being mapped match the location where it was mapped.) Only one address was identified as being incorrectly mapped. The point was deleted and the correct address was manually geocoded.

Park addresses from the three counties were obtained using the Internet and by contacting each county's parks and recreation department. Internet search terms included "parks in New Hanover County North Carolina," and returns included sites such as New Hanover County Parks (24) and the Insider's Guide to North Carolina's Southern Coast and Wilmington (25). Although larger park size is associated with increased use (26), all parks, regardless of size, were geocoded because small parks may be part of an aesthetically pleasing neighborhood environment. State-, county-, or city-maintained miniparks, neighborhood parks, community parks, large urban parks, sports complexes, and natural resource areas were included (27). Before the WISEWOMAN intervention trial began, qualitative interviews were conducted with women from the study area to learn about community factors related to PA and nutrition (28). In the qualitative interviews, women mentioned driving to use PA resources. Thus, the street addresses for all parks were geocoded, as well as additional parking areas for large parks. A total of 107 park points were geocoded, representing 102 parks.

Gyms and recreation centers in each county were identified using the Internet and by requesting information from parks and recreation departments and the New Hanover County Department on Aging. Fifty-seven gyms and recreation centers were mapped, which included 19 city- or county-funded centers (city gyms, community centers, senior centers) and 38 commercial, fee-for-use gyms.

A list of all public schools (n = 65) in the three counties was obtained from the Internet (29). Each of the 65 schools was contacted to determine whether the general public was allowed to use its PA facilities. Facilities available for public use at schools included open fields and tracks. Because only the schools that allowed public use were expected to influence PA, these were the only ones geocoded. One university, six high schools, four middle schools, and 11 elementary schools were geocoded, for a total of 22 schools.

Triangulation of data sources has been suggested to further explore quantitative associations between environmental variables and PA (30). Therefore, qualitative interviews were conducted by the first author and reviewed to identify PA resources women reported currently or previously using and PA resources they reported that a friend or relative used. Participants in the qualitative study (n = 28) were not enrolled in the WISEWOMAN Project but lived in southeastern North Carolina and were similar to WISEWOMAN participants in age and socioeconomic status (31). Because of the similarities among the women, we assumed that resources identified in the qualitative interview were resources that WISEWOMAN participants would be most likely to use. Study participants were recruited by contacting key informants from a local senior center, two community health centers, and a community recreation center. Key informants then asked women who worked at or used the centers if they would be interested in participating in the interviews. Resources reported by women were geocoded and included two school tracks, one mall, four city- or county-funded recreation centers, and eight parks. (The tracks, recreation centers, and parks included in this layer were a subset of those geocoded as described above.)

### Objectively measured proximity

The distance from each participant's home address to the closest PA resource along the road network was calculated using the ArcGIS Network Analyst extension. The number of each type of PA resource in 1- and 2-mile Euclidean ("as the crow flies") buffers was calculated using the Network Analyst intersect tool. Attribute tables generated in ArcGIS were exported into Excel and then imported into SAS Version 8.2 (SAS Institute, Cary, NC) for further analyses.

### Objectively measured MVPA

MVPA was measured using the ActiGraph accelerometer (ActiGraph, LLC, Fort Walton Beach, Fla), a valid and reliable measure of PA (32,33). Participants were instructed to wear the accelerometer for 7 consecutive days (with wear time starting the day after the clinic visit) during all waking hours. The ActiGraph measures uniaxial acceleration over investigator-specified time intervals, or epochs (1 minute in this study). The ActiProcess data reduction program (Catellier, 2004) was used to determine valid wearing time and to generate variables for use in subsequent analyses. Epochs contained within strings of 20 or more minutes of consecutive zeros were eliminated because we assumed the accelerometer was not worn. The minimum criterion for days worn was 4 days. A minimum of 6 valid hours defined a valid day: 78% (184/236) of the cohort had valid PA data. The average wearing time for the 184 participants was 11.2 hours per day and 5.7 days worn.

Average minutes of MVPA were calculated by dividing the sum of total MVPA minutes by the total number of days the accelerometer was worn. Minutes of MVPA were generated by imposing count cutpoints from Swartz et al (34), where moderate PA was 574 to 4944 accelerometer counts per minute and vigorous PA was greater than or equal to 4945 counts per minute. These cutpoints were used because participants in the Swartz et al (34) study were the most similar to WISEWOMAN participants in body mass index (BMI) and age, and the activities performed by study participants were similar to those likely to be performed by WISEWOMAN participants at a park, gym, or school.

### Covariates

Urbanicity was a potential effect modifier in the current analyses (35,36). If a participant reported a Wilmington address, she was categorized as urban; if she lived in a surrounding smaller town, she was categorized as rural. Randomization group status was examined as a covariate because the WISEWOMAN intervention aimed to increase participants' PA. The following additional covariates were examined because of their previously established independent association with PA or because they were potential confounders of the relationship between proximity (objectively measured and perceived) to resources and PA: age in years, calculated from self-reported birth date (continuous); BMI, measured at baseline as weight in kilograms divided by height in meters squared (continuous); education (dichotomized by high school graduation); baseline self-reported annual household income (greater than or equal to $10,000 and less than $10,000); self-reported race (white and nonwhite); and smoking status at baseline (dichotomous). A dichotomous variable that indicated whether participants had medical problems that limited their ability to walk briskly was also examined as a potential covariate.

### Statistical analyses

Of the 236 WISEWOMAN participants, geocoded data were collected on 199. Women whose addresses were not geocoded were generally similar to women whose addresses were geocoded, yet they were more likely to live in a rural area and lived significantly farther from the health center. As mentioned above, 184 had valid accelerometer data. Women without valid accelerometer data were significantly younger and lived farther from the health center. Of the 180 women who completed the survey containing perceived distance questions, 100 answered all three perceived distance questions. Participants with incomplete perceived distance data were significantly older and more likely to have an annual household income of less than $10,000. Therefore, the final sample was 123 women for the analyses examining 1) the correlation between perceived and objectively measured distance and 2) the association between MVPA minutes and perceived and objectively measured distance to resources. The final sample was 155 women for the analyses examining 1) agreement between perceived and objectively measured existence of resources in the neighborhood and 2) associations between MVPA minutes and resources in the neighborhood.

For the continuous variables of distance in miles, the intraclass correlation coefficient (ICC) and Pearson correlation coefficient (*r*) were used to assess agreement between GIS network distance to closest PA resource and perceived distance to the closest resource. Correlation coefficients were classified as small (0.10–0.29), moderate (0.30–0.50), and large (>0.50) (37). A SAS macro written by Robert Hamer (38) was used to calculate the ICC for perceived and objectively measured distance. There were two raters of interest: 1) women’s perceptions of distance and 2) GIS network distance. These two raters were not chosen from a larger group of possible raters and were the only raters of interest; thus, they were considered fixed effects. The paired observations of perceived and objectively measured distance were obtained from a larger population of potential WISEWOMAN participants; thus, they were considered random effects. Therefore, the two-way mixed model (ICC 3,1 in the output) was used because the raters (women’s perceptions and GIS distance) were fixed effects, and the ratings of distance were random effects.

For the dichotomous variables indicating presence or absence of resources, the kappa (κ) statistic and percentage agreement were used to assess agreement between perceived and objectively measured existence of PA resources in the neighborhood. Differences in agreement were separated into six categories: 1) less than chance agreement (κ ≤0); 2) slight agreement (κ = 0.01–0.20); 3) fair agreement (κ = 0.21–0.40); 4) moderate agreement (κ = 0.41–0.60); 5) substantial agreement (κ = 0.61–0.80); and 6) almost perfect agreement (κ = 0.81–0.99) (39). All correlations and measures of agreement were calculated for the total sample. They were also calculated separately for urban and rural participants and for participants with MVPA minutes above and below the median. Previous work suggests that perceptions of the neighborhood environment may differ based on urbanicity (40) or PA levels (22). Correlations and measures of agreement were also stratified by intervention group because the intervention included tools designed to increase awareness of PA and nutrition resources

To examine the association between MVPA minutes per day and perceived and objectively measured proximity to PA resources, the following independent variables were used: 1) GIS network distance to the closest resource, 2) perceived distance to the closest resource, 3) number of resources in GIS buffers, and 4) perceived existence of resources in the neighborhood. An additional variable was generated from the data gathered during the qualitative interviews (New Hanover County participants only, n = 132): GIS network distance to the closest PA resource. Because MVPA minutes were skewed, the natural logarithm of MVPA minutes was used as the continuous dependent variable. The association between urbanicity and GIS network distance was examined by comparing mean distance to each type of resource for urban participants compared with rural participants. Because urbanicity was associated with GIS network distance, it was not examined as a moderator (41). Multiple linear regression was used to estimate the associations between independent variables and the natural logarithm of MVPA minutes, adjusting for individual-level covariates. Covariates were retained in the final model if they had either an independent relationship with MVPA (*P* value for the parameter estimate <.05) or were confounders (changed the parameter estimate for the independent variable ≥10%). Model parsimony was a goal.

## Results

The GIS network distance to the closest PA resource by participants' urban/rural status is shown in Table 1. The mean distance to each type of resource was greater for women with rural addresses compared with women with urban addresses. Table 2 shows participant characteristics for women whose addresses were geocoded and who had valid accelerometer data. Participants' average age was 53 years and average BMI was 31 kg/m^2^; most participants were urban (77%), high school graduates (79%), nonsmokers (76%), and had annual incomes of $10,000 or more (62%). Participants averaged 112.8 minutes of MVPA per day (112.2 minutes of moderate-intensity PA per day and 0.6 minutes of vigorous-intensity PA per day). Table 2 also shows that participants tended to overestimate the distance to the closest PA resource for each type of resource.

### Correlation between perceived and objectively measured proximity

Table 3 reports agreement between perceived and objectively measured distance to closest PA resources. Pearson correlations between perceived and objectively measured distance were moderate to large for overall categories, ranging from 0.40 to 0.54. ICCs ranged from moderate for parks (0.33) to small for gyms (0.23) and schools (0.20). Correlations were generally higher for rural participants and for women in the intervention group.

Table 4 shows *κ* coefficients and percentage agreement for perceived and objectively measured existence of PA resources in 1- and 2-mile buffers. There was slight to fair agreement between the perceived and objective measures. *κ* coefficients for all participants in the 1-mile buffer category ranged from 0.14 for gyms and 0.15 for schools to 0.39 for parks. Most disagreement for parks and gyms resulted from women who reported no park or gym in the neighborhood when the GIS measure indicated one in the buffer representing the neighborhood. However, for rural women, most disagreement occurred when the woman perceived existence of a park or gym in her neighborhood when the GIS measure indicated none. Most disagreement for schools arose from women who reported a school in the neighborhood when the GIS measure indicated none. In general, rural participants had higher *κ* coefficients and percentage agreement compared with urban participants. For parks and gyms, the GIS measures using the 1-mile buffer yielded greater agreement compared with measures using the 2-mile buffer. For schools, there was greater agreement using the 2-mile buffer compared with the 1-mile buffer.

### Association between MVPA and perceived and objectively measured proximity

Table 5 shows associations between MVPA minutes per day and two measures of proximity: 1) distance to the closest PA resources, models 1 through 12; and 2) density of PA resources within 1-mile buffers, models 13 through 24. For each type of resource, the first model in the set includes the objective measure of proximity, the second model includes the perceived measure, and the third model includes both perceived and objective measures of proximity.

Models 1 through 12 in Table 5 report crude and adjusted standardized parameter estimates for the cross-sectional associations between natural logarithm-transformed MVPA minutes and perceived and objectively measured distance to the closest resources. The only statistically significant association was for perceived distance to gyms when both perceived and objectively measured distance were included in the model. Perceived distance to gyms and schools had greater parameter estimates compared with objectively measured distance, so that greater perceived distance was associated with less MVPA. Final models adjusted for age and BMI accounted for 8% to 15% variation in MVPA.

Models 13 through 24 in Table 5 show crude and adjusted standardized parameter estimates of cross-sectional associations between natural logarithm-transformed MVPA minutes and perceived existence of and objectively measured number of resources within 1-mile buffers. In all models, the number of resources in the buffer was inversely related to MVPA, against the expectation that a greater number of facilities would be associated with more activity. There was a statistically significant association between the number of schools within the 1-mile buffer and minutes of MVPA (model 19, *P* = .04; model 21, *P* = .03). Of two women of the same age (53 years) and BMI (31 kg/m^2^), one woman with no school within her 1-mile buffer averaged 105.3 minutes of MVPA per day while the other woman with two schools within her 1-mile buffer averaged 83.2 minutes of MVPA per day (*P* = .04). Variance explained by the models adjusted for age and BMI ranged from 8% to 11%.

The amount of time that women wore the accelerometer influenced the quality of PA data obtained. Thus, we examined differences in wear time by individual characteristics. We found that women who wore the accelerometer all 7 days had a lower average BMI than women who wore it 4 to 6 days (*P* = .006, data not shown). We stratified the two models in Table 5 that demonstrated statistically significant associations (i.e., models 6 and 19) by days the accelerometer was worn. Stratifying by days worn had no effect on the association between perceived distance to gyms and MVPA minutes. The association between number of schools within the 1-mile buffer and MVPA minutes was stronger and statistically significant for women who wore the accelerometer for 7 days (standardized parameter estimate = −0.38, *P* = .01, n = 44) compared with women who wore it 4 to 6 days (standardized parameter estimate = −0.08, *P* = .36, n = 111).

There was no association between distance to resources identified through qualitative interviews and MVPA minutes, adjusting for age and BMI (standardized parameter estimate for GIS network distance = 0.06, *P* = .45).

## Discussion

We measured the correlation between perceived and objectively measured distance to the closest PA resource, finding Pearson correlation coefficients of 0.40 for gyms and schools and 0.54 for parks. These findings are consistent with previous study findings that self-reported distance to a community trail and GIS network distance to the trail were significantly correlated (*r* = 0.46) (10). In our analyses, correlations were higher among rural women, perhaps because they perceive distances more accurately due to frequently driving to more urban areas. Correlations between perceived and objectively measured distance were higher for women in the intervention group than for women in the control group, possibly due to tools used in the WISEWOMAN intervention to increase participants' awareness and use of local resources. On average, perceived distance to the closest resource was greater than GIS-measured distance (Table 2), perhaps because participants were unaware of existing resources or did not perceive that a resource was accessible due to high cost or other barriers to use. Our results suggest that continued use of tools to increase women's awareness of resources may be beneficial in future WISEWOMAN interventions. Novel, multilevel strategies to help women overcome barriers to resource use are also needed.

There was slight to fair agreement between perceived existence of PA resources in the neighborhood and GIS-measured existence of resources within 1- and 2-mile buffers. Kirtland et al (22) reported fair agreement for neighborhood availability of public recreation facilities (*κ* = 0.30), with lower agreement among inactive (*κ* = 0.16) than active respondents (*κ* = 0.35). In our study, we did not find that values for *κ*were consistently higher among more active participants. However, rural participants generally had greater agreement than urban participants. This greater agreement may have resulted because few resources were located in rural areas; most rural women reported no resources in their neighborhoods, and the corresponding GIS measure indicated the same.

One might speculate that disagreement between perceived and objectively measured existence of resources in the neighborhood resulted from participants reporting proximity only to resources they would consider using (e.g., parks where participants felt safe exercising, gyms that participants could afford). Another potential reason for disagreement is that participants had different perceptions of where neighborhood boundaries were relative to investigator-defined boundaries. In addition, because one can drive much farther in 10 minutes in rural areas than in urban areas and because rural residents likely perceive their neighborhoods differently than urban residents (40), objectively measured neighborhood boundaries should be different depending on area and resident characteristics. We could have used the method of Coulton et al (40) to ask each woman to map out what she considered to be her neighborhood and then to use an average of all women's maps to create an objective (GIS) measure. Although this method would be difficult with a large sample, resident-defined boundaries may more accurately capture neighborhood exposures (42). Investigator-defined neighborhood boundaries may have limited these analyses. In addition, although proximity and accessibility measures developed using resident-defined boundaries may be the most helpful, Cho (43) found that accessibility measures derived from resident-defined boundaries were not appreciably different from accessibility measures derived from census boundaries. An additional limitation of the current study is that Euclidean buffers were used rather than road network buffers, which may offer a more precise measure of women's exposure to PA resources.

In our study, women from rural areas were farther from resources on average than urban women, consistent with qualitative and quantitative research indicating that women in rural areas have less geographic access to PA resources (44-47). This research suggests the need to increase the availability of PA resources for WISEWOMAN participants in rural areas. Disparate proximity to PA resources may be one factor contributing to lower levels of PA among rural populations compared with urban populations. WISEWOMAN participants in areas with high land-use mix and more fitness facilities had significantly lower BMI and coronary heart disease risk (48); the disparity in access to PA resources suggests a potentially serious detrimental health impact.

Healthy levels of PA can be achieved and supported through the use of existing resources such as walking trails or school tracks. Previous studies have found associations between perceived accessibility to PA resources and activity levels (7,8). In our study, perceived distance to gyms and MVPA were associated; lower perceived distance was associated with more MVPA, adjusted for age, BMI, and GIS network distance to the closest gym. This result is in agreement with other findings that perceived and objectively measured proximity (10,11,15) are associated with MVPA. There was a statistically significant, negative association between number of schools within 1-mile GIS buffers and MVPA. It is counterintuitive that more schools in the neighborhood were associated with less MVPA. This finding is perhaps due to schools often being located in areas less conducive to walking (18), with more traffic and less pedestrian infrastructure, and should be confirmed in future analyses using larger samples.

Another limitation of our study is that it was a cross-sectional analysis, so causality cannot be assumed. For example, more active women may perceive that gyms are closer because they frequently walk in the neighborhood and thus are exposed to the community environment. The study sample was small and select, thus limiting the ability to generalize results and the statistical power to detect an effect. The survey questions were not tested for reliability or validity. However, the questions have face validity and are similar to other questions that have adequate reliability. Although they may require respondents to use an element of judgment, the questions about resources where women "could exercise" are most relevant to the question of whether or not proximity to resources is associated with PA, and help take into account women's barriers to using resources.

It is possible that some information on women's proximity to PA resources was lost, because only the distance to the closest PA resource was used (not distance to all resources). In the future, multilevel modeling strategies using distance to all resources for each participant would minimize loss of data. Because we expected that only the schools that allow public access to school PA facilities would influence women's activity, only those schools were geocoded. Thus, another limitation is that we measured women's perceptions of distance to the closest school regardless of access, which may explain the lack of association with MVPA. Additionally, the low to moderate correlation between objectively measured distance and perceived distance to schools may have resulted from using an objective measure of distance to only schools that allowed public access, while the corresponding perceived distance question asked about distance to any school, regardless of access. Finally, while accelerometers provide an objective measure of PA, they cannot distinguish between types of activity, such as leisure or transportation activity.

This study had several sources of missing data. Women whose home addresses were not geocoded were more likely to be rural and to live farther from the health center than women who were geocoded. Participants with incomplete accelerometer data also lived farther from the health center. Inclusion of women who wore the accelerometer for fewer days may have introduced error into the PA measure, decreasing the likelihood of detecting statistically significant associations, because the relationship between number of schools within the 1-mile buffer and minutes of MVPA was stronger for women who wore the accelerometer 7 days compared with women who wore it fewer days.

One limitation to the methods was the fact that trails were not geocoded; however, most trails reported as walking areas by women were contained in parks, which were geocoded. The small sample could be considered a limitation, but it could also be considered a strength because it allowed collection of detailed, individual-level data. Additionally, these analyses were conduced in an understudied population of under- and uninsured women, who may have fewer resources to overcome PA barriers. Results indicate that both individual and environmental intervention strategies are needed for future WISEWOMAN interventions. One potentially effective individual-level strategy is to increase participants' awareness of existing resources. This has been achieved previously using community resource directories, which listed local PA opportunities and resources (20,49,50). Environmental strategies include enhancing access to PA resources (e.g., supplementing gym fees) or decreasing barriers to use of existing resources (e.g., increasing police patrolling in parks). More work is needed to determine the most effective individual and environmental-level strategies that will result in increased PA in low-income, midlife women.

## Figures and Tables

**Table 1:** Mean GIS Network Distance (Miles) to Closest Physical Activity Resource by Urban and Rural Status for WISEWOMAN Participants, North Carolina, 2003–2004

	Mean Distance to Closest Park	Mean Distance to Closest Gym	Mean Distance to Closest School
Urban^a^ participants (n = 147)	1.0	1.3	1.8
Rural^b^ participants (n = 52)	3.5	4.6	5.7
Total (N = 199)	1.6	2.2	2.8

GIS indicates geographic information systems.

a Participants were categorized as urban if they reported a Wilmington, NC, mailing address.

b Participants were categorized as rural if they reported an address other than Wilmington, NC.

**Table 2:** Participant (n = 155)^a^ Characteristics and Perceived and Objectively Measured Distance to Closest Physical Activity Resource, WISEWOMAN, North Carolina, 2003–2004

Characteristic	Mean Value
Age, y (SD)	53.3 (6.9)
Body mass index, kg/m^2^ (SD)	31.0 (7.8)
Mean MVPA, min (SD)	112.8 (59.0)
Mean total days accelerometer was worn (SD)	5.7 (1.1)
Mean distance from health center, miles (SD)	8.8 (8.2)
Urban^b^, %	77.4
High school graduate, %	78.7
White, %	55.5
Nonsmoker, %	76.1
Annual income^c^ <$10,000, %	37.6
Intervention group, %	54.8
No car, %	12.3
GIS network distance to closest park, miles (SD)	1.5 (1.8)
Perceived distance to closest park, miles (SD)	3.6 (4.9)
No. of parks within 1-mile GIS buffer (SD)	3.3 (3.9)
GIS network distance to closest gym, miles (SD)	2.1 (2.0)
Perceived distance to closest gym, miles (SD)	5.0 (7.0)
No. of gyms within 1-mile GIS buffer (SD)	1.1 (1.3)
GIS network distance to closest school, miles (SD)	2.6 (2.4)
Perceived distance to closest school, miles (SD)	4.9 (10.5)
No. of schools within 1-mile GIS buffer (SD)	0.5 (0.7)

GIS indicates geographic information systems; MVPA, moderate- to vigorous-intensity physical activity.

a Number includes only participants with geocoded addresses and valid accelerometer data.

b Participants were categorized as urban if they reported a Wilmington, NC, mailing address.

c Income data for six participants are missing.

**Table 3:** Agreement Between Perceived and Objectively Measured Distance to Closest Physical Activity (PA) Resource by Participant Characteristic, Overall and Stratified, WISEWOMAN, North Carolina, 2003–2004

PA Resource by Participant Characteristic	No. of Respondents^a^	Pearson *r*	Intraclass Correlation Coefficient (ICC)
Parks overall	123	0.54	0.33
Urban^b^	97	0.26	0.12
Rural^c^	26	0.60	0.40
MVPA at or above median	62	0.37	0.15
MVPA below median	61	0.75	0.60
Intervention group	67	0.61	0.36
Control group	56	0.47	0.31
Gyms overall	120	0.40	0.23
Urban^b^	90	0.13	0.04
Rural^c^	30	0.27	0.15
MVPA at or above median	60	0.35	0.22
MVPA below median	60	0.42	0.23
Intervention group	69	0.58	0.41
Control group	51	0.24	0.10
Schools overall	79	0.40	0.20
Urban^b^	57	0.18	0.11
Rural^c^	22	0.27	0.11
MVPA at or above median	40	0.54	0.47
MVPA below median	39	0.39	0.17
Intervention group	46	0.44	0.36
Control group	33	0.42	0.17
All resources overall	74	0.61	0.34
Urban^b^	54	0.26	0.14
Rural^c^	20	0.52	0.25
MVPA at or above median	37	0.65	0.43
MVPA below median	37	0.59	0.31
Intervention group	44	0.82	0.54
Control group	30	0.49	0.25

MVPA indicates moderate- to vigorous-intensity PA.

a Not all numbers total 123 because participants with missing GIS and accelerometer data were not included in the analysis.

b Participants were categorized as urban if they reported a Wilmington, NC, mailing address.

c Participants were categorized as rural if they reported an address other than Wilmington, NC.

**Table 4:** Agreement Between Perceived and Objectively Measured Existence of Physical Activity Resources in 1- and 2-mile Buffers, Overall and Stratified, WISEWOMAN Participants (n = 155), North Carolina, 2003–2004

Participant Characteristics	No. of Respondents	Gym		Park		School	

		*κ* (95% CI)	% Agreement	*κ* (95% CI)	% Agreement	*κ* (95% CI)	% Agreement
**1-mile buffer**							
All participants	155	0.14 (−0.02 to 0.29)	57.4	0.39 (0.24 to 0.53)	70.6	0.15 (0.01 to 0.28)	54.8
Urban^a^	120	0.07 (−0.10 to 0.24)	52.5	0.31(0.14 to 0.48)	70.3	0.08 (−0.09 to 0.23)	51.7
Rural^b^	35	0.26 (−0.08 to 0.60)	74.3	0.31 (−0.02 to 0.64)	71.4	0.24 (−0.03 to 0.50)	65.7
MVPA at or above median	78	0.14 (−0.08 to 0.35)	57.7	0.39 (0.18 to 0.60)	71.0	0.11 (−0.06 to 0.27)	50.0
MVPA below median	77	0.14 (−0.08 to 0.35)	57.1	0.39 (0.19 to 0.59)	70.1	0.21 (0.00 to 0.42)	59.7
Intervention group	85	0.29 (0.09 to 0.49)	64.7	0.37 (0.17 to 0.56)	69.0	0.08 (−0.09 to 0.25)	49.4
Control group	70	−0.05 (−0.28 to 0.19)	48.6	0.42 (0.20 to 0.63)	72.5	0.25 (0.03 to 0.46)	61.4
**2-mile buffer**							
All participants	155	0.09 (0.00 to 0.19)	47.7	0.16 (0.04 to 0.27)	61.4	0.18 (0.02 to 0.34)	62.6
Urban^a^	120	−0.02 (−0.08 to 0.05)	40.8	0.02 (−0.07 to 0.11)	61.9	0.03 (−0.14 to 0.20)	62.5
Rural^b^	35	0.34 (0.01 to 0.67)	71.4	0.23 (−0.05 to 0.52)	60.0	0.19 (−0.11 to 0.50)	62.9
MVPA at or above median	78	0.10 (−0.04 to 0.23)	47.4	0.06 (−0.09 to 0.22)	59.2	0.23 (0.00 to 0.45)	65.4
MVPA below median	77	0.08 (−0.06 to 0.23)	48.1	0.24 (0.07 to 0.41)	63.6	0.14 (−0.08 to 0.35)	59.7
Intervention group	85	0.07 (−0.06 to 0.20)	45.9	0.12 (−0.03 to 0.27)	58.3	0.13 (−0.09 to 0.35)	61.2
Control group	70	0.11 (−0.04 to 0.26)	50.0	0.21 (0.03 to 0.40)	65.2	0.23 (0.00 to 0.46)	64.3

CI indicates confidence interval; MVPA, moderate- to vigorous-intensity physical activity.

a Participants were categorized as urban if they reported a Wilmington, NC, mailing address.

b Participants were categorized as rural if they reported an address other than Wilmington, NC.

**Table 5:** Estimates of Associations Between Natural Logarithm of Minutes of MVPA and Perceived and Objectively Measured Distance to Closest Physical Activity (PA) Resource and Density of Physical Activity Resources, WISEWOMAN, North Carolina, 2003–2004

Model No.^a^	Independent Variable(s)	No. of Respondents	Crude Standardized Parameter Estimate	*P*	Adjusted *R^2^*	Adjusted Standardized Parameter Estimate	*P*	Adjusted *R^2^*
**Distance to closest PA resource**								
To closest park								
1	GIS network distance^b^	129	−0.06	.53	−0.01	−0.05	.56	0.10
2	Perceived distance^b^	123	0.01	.92	−0.01	0.02	.81	0.10
3	GIS network distance^b^	123	−0.06	.61	−0.01	−0.07	.53	0.09
	Perceived distance^b^		0.04	.72		0.06	.58	
To closest gym								
4	GIS network distance^b^	129	0.08	.39	−0.002	0.05	.54	0.10
5	Perceived distance^b^	120	−0.17	.06	0.02	−0.13	.12	0.14
6	GIS network distance^b^	120	0.18	.07	0.04	0.13	.18	0.15
	Perceived distance^b^		−0.24	.01		−0.19	.05	
To closest school								
7	GIS network distance^b^	129	0.07	.42	−0.003	0.06	.49	0.10
8	Perceived distance^c^	79	−0.20	.08	0.03	−0.18	.09	0.11
9	GIS network distance^b^	79	0.08	.51	0.02	0.09	.43	0.10
	Perceived distance^b^		−0.23	.07		−0.22	.06	
To closest of combination								
10	GIS network distance^b^	129	0.04	.66	−0.01	0.03	.76	0.10
11	Perceived distance^b^	74	−0.16	.18	0.01	−0.15	.19	0.09
12	GIS network distance^b^	74	0.12	.40	0.01	0.08	.56	0.08
	Perceived distance^b^		−0.23	.12		−0.20	.16	
**Objectively measured no. and perceived existence of PA resources in neighborhood^d^**								
Parks in neighborhood								
13	No. of parks in buffer	155	−0.08	.34	0.00	−0.06	.41	0.08
14	Perceived existence of parks	155	0.11	.18	0.01	0.08	.34	0.09
15	No. of parks in buffer	155	−0.09	.25	0.01	−0.08	.34	0.09
	Perceived existence of parks		0.12	.14		0.09	.28	
Gyms in neighborhood								
16	No. of gyms in buffer	155	−0.10	.24	0.00	−0.06	.44	0.08
17	Perceived existence of gyms	155	−0.02	.80	−0.01	−0.04	.60	0.08
18	No. of gyms in buffer	155	−0.09	.24	0.00	−0.06	.46	0.08
	Perceived existence of gyms		−0.02	.83		−0.04	.63	
Schools in neighborhood								
19	No. of schools in buffer	155	−0.16	.04	0.02	−0.16	.04	0.11
20	Perceived existence of schools	155	0.07	.40	0.00	0.04	.61	0.08
21	No. of schools in buffer	155	−0.18	.03	0.02	−0.17	.03	0.10
	Perceived existence of schools		0.09	.26		0.06	.42	
Combination of all resources								
22	No. of all resources in buffer	155	−0.11	.16	0.01	−0.09	.23	0.09
23	Perceived existence of all resources	155	0.08	.32	0.00	0.04	.64	0.08
24	No. of all resources in buffer	155	−0.13	.11	0.01	−0.10	.19	0.09
	Perceived existence of all resources		0.10	.21		0.06	.48	

GIS indicates geographic information systems.

a For each type of resource, the first model in the set includes the objective measure of proximity, the second model includes the perceived measure of proximity, and the third model includes both perceived and objective measures of proximity.

b Adjusted estimates include age and body mass index (BMI). Other covariates (intervention group, race, education, income, smoking, and PA limitations) were not independently associated with activity and were not significant confounders.

c Adjusted for body mass index only.

d Neighborhood was defined objectively as a 1-mile GIS buffer.
